# Refining trigonometric inequalities by using Padé approximant

**DOI:** 10.1186/s13660-018-1742-7

**Published:** 2018-06-27

**Authors:** Zhen Zhang, Huaqing Shan, Ligeng Chen

**Affiliations:** 1 School of Cyberspace, Hangzhou Dianzi University, Hangzhou, China; 2 Key Laboratory of Complex Systems Modeling and Simulation, Hangzhou Dianzi University, Hangzhou, China

**Keywords:** Trigonometric inequalities, Padé approximant, Two-sided bounds, Rational refinement

## Abstract

A two-point Padé approximant method is presented for refining some remarkable trigonometric inequalities including the Jordan inequality, Kober inequality, Becker–Stark inequality, and Wu–Srivastava inequality. Simple proofs are provided. It shows to achieve better approximation results than those of prevailing methods.

## Introduction

Trigonometric inequalities have caused interest of a lot of researchers, they analyzed the Wilker inequality [6–11, 14, 16–19], Jordan inequality [3, 5, 15, 20, 21], Shafer–Fink inequality [12], Becker–Stark inequalities [13], and so on.

Recently, Bercu provided a Padé-approximant-based method and obtained the following inequalities [2].

1 $$\begin{aligned} &b_{1}(x)= \frac{-7 x^{2} + 60}{3 x^{2} + 60} < \frac{\sin(x)}{x} < \frac{11 x^{4} - 360 x^{2}+ 2520}{60 x^{2}+ 2520}= b_{2}(x),\quad \forall x \in (0,\pi/2); \end{aligned}$$

2 $$\begin{aligned} & \begin{aligned}[b] b_{3}(x) &=\frac{17x^{4} - 480x^{2}+ 1080}{2x^{4}+ 60x^{2}+ 1080} < \cos(x) < \frac{3x^{4} - 56x^{2}+ 120}{4x^{2}+ 120} \\ &= b_{4}(x),\quad \forall x \in (0,\pi/2); \end{aligned} \end{aligned}$$

3 $$\begin{aligned} & b_{5}(x) < \frac{\tan(x)}{x}< b_{6}(x),\quad \forall x \in (0,1.5701); \end{aligned}$$

4 $$\begin{aligned} & \biggl(\frac{x}{\sin(x)}\biggr)^{2}+\frac{x}{\tan(x)} > b_{7}(x),\quad \forall x \in (0,1.5701), \end{aligned}$$

where $b_{5}(x)=\frac{-28x^{4} - 600x^{2}+ 7200}{9x^{6}+ 12x^{4} - 3000x^{2}+ 7200}$, $b_{6}(x)=\frac{22x^{8} - 60x^{6} - 4680x^{4}- 237\mbox{,}600x^{2}+ 2\mbox{,}721\mbox{,}600}{1020x^{6}+ 14\mbox{,}040x^{4}- 1\mbox{,}144\mbox{,}800x^{2}+ 2\mbox{,}721\mbox{,}600}$ and $b_{7}(x)=\frac{11\mbox{,}220x^{10}-205\mbox{,}560x^{8}-14\mbox{,}256\mbox{,}000x^{6}+512\mbox{,}179\mbox{,}200x^{4}- 3\mbox{,}157\mbox{,}056\mbox{,}000x^{2}+13\mbox{,}716\mbox{,}864\mbox{,}000}{242x^{12}-8580x^{10} +25\mbox{,}560x^{8}-1\mbox{,}080\mbox{,}000x^{6}+103\mbox{,}680\mbox{,}000x^{4}-1\mbox{,}578\mbox{,}528\mbox{,}000x^{2}+6\mbox{,}858\mbox{,}432\mbox{,}000}$.

In this paper, we present a two-point Padé-approximant-based method [1] for refining the rational bounds of several trigonometric inequalities, and also provide a method for proving the refined bounds. By applying the new method to $\frac{\sin (x)}{x}$ and $\cos(x)$, we refine the bounds of Eq. (1) ∼ (2), for $\forall x \in [0,\pi/2]$, see also Theorems 3.1 and 3.2. Applied to $\frac{\tan (x)}{x}$ and $(\frac{x}{\sin(x)})^{2}+\frac{x}{\tan(x)}$, it not only provides refined two-sided bounds with better approximation effect for Eq. (3) ∼ (4), but also extends the interval $(0,1.5701)$ to the interval $[0,\pi/2]$, see also the theorems and remarks in Sect. 3.

## Find bounds by using two-point Padé approximant

Given a bounded smooth function $f(x)$, $x \in [x_{0}, x_{1}]$, let $R(x)=\frac{\sum^{n}_{i=0} c_{i} x^{i}}{1+\sum^{m}_{i=1} d_{i} x^{i}}$ be a rational polynomial interpolating derivatives of $f(x)$ at two points $x_{0}$ and $x_{1}$ such that

5 $$ E^{(i)}(x_{0})=0, \qquad E^{(j)}(x_{1})=0,\quad i=0,1, \ldots,k, j=0,1,\ldots,l, $$

where $E(x)=(1+\sum^{m}_{i=1}d_{i} x^{i}) \cdot f(x) -(\sum^{n}_{i=1}c_{i} x^{i})$. There are $m+n+2$ unknowns in Eq. (5). By selecting suitable values of *k* and *l*, we have that Eq. (5) consists of $m+n+2$ linear equations in the unknown variables $c_{i}$ and $d_{j}$, and the interpolation polynomial $R(x)$ can be determined by solving Eq. (5).

We give two examples. Without loss of generality, let $\Gamma=[0,\pi/2]$.

### Example 1

Let $f_{1}(x)=\sin(x)$. By setting $n_{1}=13$, $m_{1}=0$, $n_{2}=11$, and $m_{2}=0$ and introducing the following constraints

6 $$ f_{1}^{(i)}(0)=R_{j}^{(i)}(0),\qquad f_{1}(\pi/2)=R_{j}(\pi/2),\quad j=1,2, i=0,1,\ldots,14-2j, $$

we obtain that

7 $$ R_{1}(x)= \beta_{1}(x)+ \alpha_{1} \cdot x^{13}, \qquad R_{2}(x)= \beta_{2}(x)- \alpha_{2} \cdot x^{11}, $$

where $\alpha_{1}=\frac{\pi^{11}-440 \pi^{9}+126\mbox{,}720 \pi^{7}-21\mbox{,}288\mbox{,}960 \pi^{5}+1\mbox{,}703\mbox{,}116\mbox{,}800 \pi^{3}-40\mbox{,}874\mbox{,}803\mbox{,}200 \pi+81\mbox{,}749\mbox{,}606\mbox{,}400}{9\mbox{,}979\mbox{,}200 \pi^{13}}$, $\beta_{1}(x)=t-\frac{t^{3}}{6}+ \frac{t^{5}}{120} - \frac{t^{7}}{5040} + \frac{t^{9}}{362\mbox{,}880}-\frac{x^{11}}{39\mbox{,}916\mbox{,}800}$, $\alpha_{2}=\frac{\pi^{9}-288 \pi^{7}+48\mbox{,}384 \pi^{5}-3\mbox{,}870\mbox{,}720 \pi^{3} +92\mbox{,}897\mbox{,}280 \pi-185\mbox{,}794\mbox{,}560}{90\mbox{,}720 \pi^{11}} $, $\beta_{2}(x)=t-\frac{t^{3}}{6}+ \frac{t^{5}}{120} - \frac{t^{7}}{5040} + \frac{t^{9}}{362\mbox{,}880}$. It can be verified that $R_{j}(x) \geq 0, \forall x \in \Gamma, j=1,2$. From Eq. (6), $\forall x \in \Gamma$, there exists $\xi_{j}(x) \in \Gamma$ such that [4]

8 $$ f_{1}(x) - R_{j}(x) = \frac{f_{1}^{(16-2j)}(\xi_{j}(x))}{(16-2j)!} \cdot (x-\pi/2) \cdot x^{15-2j}, \quad x \in \Gamma, j=1,2. $$

Note that $f_{1}^{(14)}(x) = -\sin(x) \leq 0$ and $f_{1}^{(12)}(x) = \sin(x) \geq 0$, $\forall x \in \Gamma$. Combining with Eq. (8), one obtains that

9 $$ 0 \leq R_{1}(x) \leq \sin(x) \leq R_{2}(x), \quad x \in \Gamma. $$

### Example 2

Let $f_{2}(x)=\cos(x)$. By setting $n_{3}=12$, $m_{3}=0$, $n_{4}=10$, and $m_{4}=0$ and introducing the following constraints

10 $$ f_{2}^{(i)}(0)=R_{j}^{(i)}(0),\qquad f_{2}(\pi/2)=R_{j}(\pi/2),\quad j=3,4, i=0,1,\ldots,17-2j, $$

we obtain that

11 $$ R_{3}(x)= \beta_{3}(x)+ \alpha_{3} \cdot x^{12} , \qquad R_{4}(x)= \beta_{4}(x)- \alpha_{4} \cdot x^{10}, $$

where $\alpha_{3}=\frac{\pi^{10}-360 \pi^{8}+80\mbox{,}640 \pi^{6}-9\mbox{,}676\mbox{,}800 \pi^{4}+464\mbox{,}486\mbox{,}400 \pi^{2}-3\mbox{,}715\mbox{,}891\mbox{,}200}{907\mbox{,}200 \pi^{12}}$, $\beta_{3}(x)=1-\frac{x^{2}}{2} +\frac{x^{4}}{24} -\frac{x^{6}}{720} +\frac{x^{8}}{40\mbox{,}320} -\frac{x^{10}}{3\mbox{,}628\mbox{,}800}$, $\alpha_{4}=\frac{10\mbox{,}321\mbox{,}920-1\mbox{,}290\mbox{,}240 \pi^{2}+26\mbox{,}880 \pi^{4}-224 \pi^{6}+\pi^{8}}{10\mbox{,}080 \pi^{10}} $, $\beta_{4}(x)=1-\frac{x^{2}}{2} +\frac{x^{4}}{24} -\frac{x^{6}}{720} +\frac{x^{8}}{40\mbox{,}320} $. It can be verified that $R_{j}(x) \geq 0, \forall x \in \Gamma, j=3,4$. From Eq. (10), $\forall x \in \Gamma$, there exists $\xi_{j}(x) \in \Gamma, j=3,4$, such that [4]

12 $$ f_{2}(x) - R_{j}(x) = \frac{f_{2}^{(19-2j)}(\xi_{j}(x))}{(19-2j)!} \cdot (x-\pi/2) \cdot x^{18-2j}, \quad x \in \Gamma, j=3,4. $$

Note that $f_{2}^{(13)}(x) = -\sin(x) \leq 0$ and $f_{2}^{(11)}(x) = \sin(x) \geq 0$, $\forall x \in \Gamma$. Combining with Eq. (12), one obtains that

13 $$ 0 \leq R_{3}(x) \leq \cos(x) \leq R_{4}(x), \quad x \in \Gamma. $$

## Main results

The main results are as follows.

### Theorem 3.1

*For all* $\forall x \in \Gamma=[0,\pi/2]$, *we have that*

14 $$\begin{aligned}{} [b] c_{1}(x) &= \frac{60\mbox{,}480-9240 x^{2}+364 x^{4}-5 x^{6} }{840 (72+x^{2})} \leq \frac{\sin(x)}{x} \\ &\leq \frac{ (166\mbox{,}320-22\mbox{,}260 x^{2}+551 x^{4}) }{15 (11\mbox{,}088+364 x^{2}+5 x^{4})} =c_{2}(x). \end{aligned}$$

### Proof

Eq. (14) is equivalent to

15 $$ \textstyle\begin{cases} (60\mbox{,}480-9240 x^{2}+364 x^{4}-5 x^{6}) x - 840 (72+x^{2}) \sin(x) \leq 0, \\ (166\mbox{,}320-22\mbox{,}260 x^{2}+551 x^{4}) x - 15 (11\mbox{,}088+364 x^{2}+5 x^{4}) \sin(x) \geq 0, \end{cases}\displaystyle \forall x \in \Gamma. $$

It is well known that $\forall x \in \Gamma$,

16 $$\begin{aligned} \beta_{1}(x)&=t-\frac{t^{3}}{6}+ \frac{t^{5}}{120} - \frac{t^{7}}{5040} + \frac{t^{9}}{362\mbox{,}880}-\frac{x^{11}}{39\mbox{,}916\mbox{,}800} \\ &\leq \sin(x) \leq \beta_{1}(x) + \frac{x^{13}}{6\mbox{,}227\mbox{,}020\mbox{,}800}. \end{aligned}$$

Combining with Eq. (16), we have that

$$\begin{aligned} &\bigl(60\mbox{,}480-9240 x^{2}+364 x^{4}-5 x^{6}\bigr) x - 840 \bigl(72+x^{2}\bigr) \sin(x) \\ &\quad \leq \bigl(60\mbox{,}480-9240 x^{2}+364 x^{4}-5 x^{6}\bigr) x - 840 \bigl(72+x^{2}\bigr) \beta_{1}(x) \\ &\quad =\frac{x^{11} \cdot (-38+x^{2})}{39\mbox{,}916\mbox{,}800} \leq 0, \quad \forall x \in \Gamma, \\ & \bigl(166\mbox{,}320-22\mbox{,}260 x^{2}+551 x^{4}\bigr) x - 15 \bigl(11\mbox{,}088+364 x^{2}+5 x^{4}\bigr) \sin(x) \\ &\quad \geq \bigl(166\mbox{,}320-22\mbox{,}260 x^{2}+551 x^{4}\bigr) x\\ &\qquad {} - 15 \bigl(11\mbox{,}088+364 x^{2}+5 x^{4}\bigr) \biggl( \beta_{1}(x) + \frac{x^{13}}{6\mbox{,}227\mbox{,}020\mbox{,}800}\biggr) \\ &\quad = \frac{x^{11}}{6\mbox{,}227\mbox{,}020\mbox{,}800} \bigl(1\mbox{,}661\mbox{,}088-40\mbox{,}104 x^{2}+ 416 x^{4}- 5 x^{6}\bigr) \\ &\quad \geq \frac{x^{11}}{6\mbox{,}227\mbox{,}020\mbox{,}800} \bigl(1\mbox{,}661\mbox{,}088-40\mbox{,}104 \cdot 2^{2} - 5 \cdot 2^{6}\bigr) \geq 0,\quad \forall x \in \Gamma, \end{aligned}$$

which is just Eq. (15). So we have completed the proof of Eq. (14). □

### Theorem 3.2

*For all* $\forall x \in [0,\pi/2]$, *we have that*

17 $$ \begin{aligned}[b] c_{3}(x)&= \frac{ 20\mbox{,}160-9720 x^{2}+ 660 x^{4}-13 x^{6}}{360 (x^{2}+56)} \leq \cos(x) \\ & \leq \frac{15\mbox{,}120-6900 x^{2}+313 x^{4}}{15\mbox{,}120+660 x^{2}+13 x^{4}} =c_{4}(x). \end{aligned} $$

### Proof

Eq. (17) is equivalent to

18 $$ \textstyle\begin{cases} (20\mbox{,}160-9720 x^{2}+ 660 x^{4}-13 x^{6})- 360 (x^{2}+56) \cos(x) \leq 0, \\ (15\mbox{,}120-6900 x^{2}+313 x^{4}) - (15\mbox{,}120+660 x^{2}+13 x^{4}) \cos(x) \geq 0, \end{cases}\displaystyle \forall x \in \Gamma. $$

It is well known that

19 $$ \begin{aligned}[b] \beta_{3}(x)&=1-\frac{x^{2}}{2} + \frac{x^{4}}{24} -\frac{x^{6}}{720} +\frac{x^{8}}{40\mbox{,}320} -\frac{x^{10}}{3\mbox{,}628\mbox{,}800} \leq \cos(x) \\ & \leq 1-\frac{x^{2}}{2} +\frac{x^{4}}{24} -\frac{x^{6}}{720} + \frac{x^{8}}{40\mbox{,}320} = \beta_{4}(x), \quad \forall x \in \Gamma. \end{aligned} $$

Combining with Eq. (19), we have that

20 $$ \textstyle\begin{cases} (20\mbox{,}160-9720 x^{2}+ 660 x^{4}-13 x^{6})- 360 (x^{2}+56) \cos(x) \\ \quad \leq (20\mbox{,}160-9720 x^{2}+ 660 x^{4}-13 x^{6})- 360 (x^{2}+56) \beta_{3}(x) \\ \quad = \frac{x^{10}}{3\mbox{,}628\mbox{,}800} (-34+x^{2}) \leq 0, \quad \forall x \in [0,\pi/2], \\ (15\mbox{,}120-6900 x^{2}+313 x^{4}) - (15\mbox{,}120+660 x^{2}+13 x^{4}) \cos(x) \\ \quad \geq (15\mbox{,}120-6900 x^{2}+313 x^{4}) - (15\mbox{,}120+660 x^{2}+13 x^{4}) \beta_{4}(x) \\ \quad =\frac{x^{10}}{40\mbox{,}320} (68 -13 x^{2}) \geq 0, \quad \forall x \in [0,\pi/2]. \end{cases} $$

Thus, we have completed the proof of both Eq. (18) and Eq. (17). □

### Theorem 3.3

*For all* $\forall x \in \Gamma$, *we have that*

21 $$ \begin{aligned}[b] c_{5}(x)&= \frac{ 21(495-60 x^{2}+x^{4})}{10\mbox{,}395-4725 x^{2}+210 x^{4}-x^{6}} \leq \frac{\tan(x)}{x} \\ &\leq \frac{T_{1}(x)}{105 (\pi^{2}-4 x^{2}) \cdot T_{2}(x)} =c_{6}(x), \end{aligned} $$

*where* $T_{1}(x)=(\pi^{6}-840 \pi^{4}+75\mbox{,}600 \pi^{2}-665\mbox{,}280) x^{6} + (210 \pi^{6}+52\mbox{,}920 \pi^{4}-7\mbox{,}620\mbox{,}480 \pi^{2}+69\mbox{,}854\mbox{,}400) x^{4} + (-17\mbox{,}955 \pi^{6}+1\mbox{,}323\mbox{,}000 \pi^{4}+52\mbox{,}390\mbox{,}800 \pi^{2}-628\mbox{,}689\mbox{,}600) x^{2} + (155\mbox{,}925 (\pi^{4}-112 \pi^{2}+1008)) \pi^{2}$ *and* $T_{2}(x) = (26 \pi^{4}-2664 \pi^{2}+23\mbox{,}760) x^{4} + (-666 \pi^{4}+73\mbox{,}980 \pi^{2}-665\mbox{,}280) x^{2} + (1485 \pi^{4}-166\mbox{,}320 \pi^{2}+1\mbox{,}496\mbox{,}880)$.

### Proof

Eq. (21) is equivalent to

22 $$ \textstyle\begin{cases} \begin{aligned} H_{5}(x) ={}& 21(495-60 x^{2}+x^{4}) \cdot x \cos(x) \\ &{} - (10\mbox{,}395-4725 x^{2}+210 x^{4}-x^{6}) \cdot \sin(x) \leq 0; \end{aligned} \\ H_{6}(x) =105 (\pi^{2}-4 x^{2}) \cdot T_{2}(x) \cdot \sin(x) -T_{1}(x) \cdot x \cos(x) \leq 0, \end{cases}\displaystyle \forall x \in \Gamma. $$

It can be verified that

23 $$ \textstyle\begin{cases} \cos(x) \leq 1-\frac{x^{2}}{2} +\frac{x^{4}}{24} -\frac{x^{6}}{720} +\frac{x^{8}}{40\mbox{,}320} -\frac{x^{10}}{3\mbox{,}628\mbox{,}800} + \frac{x^{12}}{479\mbox{,}001\mbox{,}600}= \beta_{5}(x), \\ \beta_{1}(x)=t-\frac{t^{3}}{6}+ \frac{t^{5}}{120} - \frac{t^{7}}{5040} + \frac{t^{9}}{362\mbox{,}880}-\frac{x^{11}}{39\mbox{,}916\mbox{,}800} \leq \sin(x), \\ 495-60 x^{2}+x^{4}>0,\qquad 10\mbox{,}395-4725 x^{2}+210 x^{4}-x^{6}>0, \end{cases}\displaystyle \forall x \in \Gamma. $$

Combining with Eq. (23), we have that

24 $$ \begin{aligned}[b] H_{5}(x) &\leq 21\bigl(495-60 x^{2}+x^{4} \bigr) \cdot x \beta_{5}(x) - \bigl(10\mbox{,}395-4725 x^{2}+210 x^{4}-x^{6}\bigr) \cdot \beta_{1}(x) \\ &=\frac{x^{13}}{159\mbox{,}667\mbox{,}200} \bigl(-915-64 x^{2}+3 x^{4}\bigr) \leq 0, \quad \forall x \in \Gamma. \end{aligned} $$

Let $\beta_{6}(x)=T_{1}(x)+105 (\pi^{2}-4 x^{2}) \cdot T_{2}'(x) -840 x \cdot T_{2}(x)$, $\beta_{7}(x)=105 (\pi^{2}-4 x^{2}) \cdot T_{2}(x)-T_{1}'(x)$. On the other hand, it can be verified that, $\forall x \in \Gamma$,

25 $$ \begin{aligned} &H_{6}'(x)= \beta_{6}(x) \cdot \sin(x) + \beta_{7}(x) \cdot \cos(x), \\ &\beta_{6}(x) \leq 0,\qquad \beta_{7}(x) \geq 0,\qquad T_{2}(x) \geq 0,\qquad T_{1}(x) \geq 0, \\ & \begin{aligned} \cos(x) \geq{}& 1-\frac{x^{2}}{2} +\frac{x^{4}}{24} -\frac{x^{6}}{720} + \frac{x^{8}}{40\mbox{,}320} -\frac{x^{10}}{3\mbox{,}628\mbox{,}800} + \frac{x^{12}}{479\mbox{,}001\mbox{,}600} \\ &{}-\frac{x^{14}}{87\mbox{,}178\mbox{,}291\mbox{,}200}= \beta_{8}(x), \end{aligned}\\ &\beta_{9}(x)=t-\frac{t^{3}}{6}+ \frac{t^{5}}{120} - \frac{t^{7}}{5040} + \frac{t^{9}}{362\mbox{,}880}-\frac{x^{11}}{39\mbox{,}916\mbox{,}800}+\frac{x^{13}}{6\mbox{,}227\mbox{,}020\mbox{,}800}\geq \sin(x). \end{aligned} $$

Combining Eq. (23) with Eq. (25), we have that

26 $$ \begin{aligned} H_{6}(x) &\leq 105 \bigl(\pi^{2}-4 x^{2}\bigr) \cdot T_{2}(x) \cdot \beta_{9}(x) -T_{1}(x) \cdot x \beta_{8}(x) \\ &= \frac{x^{13}}{9\mbox{,}153\mbox{,}720\mbox{,}576\mbox{,}000} \beta_{10}(x) \leq 0, \quad \forall x \in \biggl[0,\frac{31 \pi}{64}\biggr], \\ H_{6}'(x) &\geq \beta_{6}(x) \cdot \beta_{1}(x) + \beta_{7}(x) \cdot \beta_{5}(x) \\ &= \frac{x^{12}}{50\mbox{,}295\mbox{,}168\mbox{,}000} \beta_{11}(x) \geq 0, \quad \forall x \in \biggl[\frac{31 \pi}{64},\frac{\pi}{2}\biggr], \end{aligned} $$

where $\beta_{10}(x)=(18\mbox{,}063\mbox{,}360 \pi^{6}-8\mbox{,}128\mbox{,}512\mbox{,}000 \pi^{4}+ 643\mbox{,}778\mbox{,}150\mbox{,}400 \pi^{2}- 5\mbox{,}579\mbox{,}410\mbox{,}636\mbox{,}800)+( -634\mbox{,}725 \pi^{6}+305\mbox{,}912\mbox{,}880 \pi^{4}-24\mbox{,}700\mbox{,}198\mbox{,}320 \pi^{2}+ 214\mbox{,}592\mbox{,}716\mbox{,}800) x^{2} +(6069 \pi^{6}-4\mbox{,}639\mbox{,}320 \pi^{4}+411\mbox{,}823\mbox{,}440 \pi^{2}-3\mbox{,}618\mbox{,}457\mbox{,}920) x^{4} + (28 \pi^{6}+52\mbox{,}920 \pi^{4}-5\mbox{,}715\mbox{,}360 \pi^{2}+51\mbox{,}226\mbox{,}560) x^{6} + (\pi^{6}-840 \pi^{4}+75\mbox{,}600 \pi^{2}-665\mbox{,}280) x^{8} \leq 0, \forall x \in [0,\frac{31 \pi}{64}]$, $\beta_{11}(x)=(-1\mbox{,}290\mbox{,}240 \pi^{6}+580\mbox{,}608\mbox{,}000 \pi^{4}-45\mbox{,}984\mbox{,}153\mbox{,}600 \pi^{2}+ 398\mbox{,}529\mbox{,}331\mbox{,}200)+ (54\mbox{,}405 \pi^{6}-25\mbox{,}552\mbox{,}800 \pi^{4}+2\mbox{,}048\mbox{,}684\mbox{,}400 \pi^{2}- 17\mbox{,}782\mbox{,}934\mbox{,}400) x^{2}+ ( -1404 \pi^{6}+556\mbox{,}920 \pi^{4}-42\mbox{,}366\mbox{,}240 \pi^{2}+365\mbox{,}238\mbox{,}720) x^{4}+ ( 19 \pi^{6}-5040 \pi^{4}+317\mbox{,}520 \pi^{2}-2\mbox{,}661\mbox{,}120) x^{6}\geq 0, \forall x \in [\frac{31 \pi}{64},\frac{\pi}{2}]$. Combining Eq. (26) with $H_{6}(\pi/2)=0$, we obtain that

27 $$ H_{6}(x) \leq 0,\quad \forall x \in \biggl[0,\frac{\pi}{2} \biggr]. $$

Combining Eq. (24) with Eq. (27), we have completed the proof of both Eq. (22) and Eq. (21). □

From Theorems 3.1, 3.2, and 3.3, we directly obtain the following theorem.

### Theorem 3.4

*We have that*

$$\frac{1}{c_{2}(x)^{2}} + \frac{1}{c_{6}(x)} \leq \biggl(\frac{x}{\sin(x)} \biggr)^{2} + \frac{x}{\tan(x)} \leq \frac{1}{c_{1}(x)^{2}} + \frac{1}{c_{5}(x)},\quad \forall x \in [0,\pi/2]. $$

## Discussion and conclusions

Firstly, we compare the results of $\frac{\sin(x)}{x}$ between $b_{i}(x)$ in [2] and $c_{i}(x)$ in this paper, $i=1,2$. It can be verified that $c_{1}(x)-b_{1}(x)=\frac{x^{6} (264-5x^{2})}{840(72+x^{2})(x^{2}+20)} \geq 0$ and $c_{2}(x)-b_{2}(x)=\frac{ -11 x^{8}}{12(11\mbox{,}088+364 x^{2}+5 x^{4})(x^{2}+42)} \leq 0$, $\forall x \in [0,\pi/2]$, we have that

$$b_{1}(x) \leq c_{1}(x) \leq \frac{\sin(x)}{x} \leq c_{2}(x) \leq b_{2}(x), \quad \forall x \in [0,\pi/2]. $$

Secondly, we compare the approximation results of $\cos(x)$ between previous $b_{i}(x)$ and present $c_{i}(x)$, $i=3,4$. It can be verified that $c_{3}(x)-b_{3}(x)=\frac{x^{8}(270-13 x^{2})}{360 (56+x^{2})(x^{4}+30 x^{2}+540)} \geq 0$ and $c_{2}(x)-b_{2}(x)=\frac{ -39 x^{8}}{4 (15\mbox{,}120+660 x^{2}+13 x^{4})(x^{2}+30)} \leq 0$, $\forall x \in [0,\pi/2]$, we have that

$$b_{3}(x) \leq c_{3}(x) \leq \cos(x) \leq c_{4}(x) \leq b_{4}(x), \quad \forall x \in [0,\pi/2]. $$

Thirdly, we compare the approximation results of $\frac{\tan(x)}{x}$, which also shows that this paper achieves a much better result. It can be verified that $\forall x \in [0,\pi/2]$,

$$c_{5}(x)-b_{5}(x)=\frac{x^{6}(161 x^{2}-495) (x^{2}-33)}{3(10\mbox{,}395-4725 x^{2}+210 x^{4}-x^{6}) (x^{2}+20) (3 x^{4}-56 x^{2}+120)} \geq 0. $$

However, note that the denominator of $b_{6}(x)$ is $T_{3}(x) = 1020x^{6}+ 14\mbox{,}040x^{4}- 1\mbox{,}144\mbox{,}800x^{2}+ 2\mbox{,}721\mbox{,}600 = 30(17 x^{4}-480 x^{2}+1080)(x^{2}+42)$, which has a real root ≈1.5701 within the interval Γ, and we have $T_{3}(x) >0, \forall x \in [0,1.5701]$. It can be verified that $c_{6}(x) - b_{6}(x)= \frac{-x^{8} H_{7}(x)}{210 T_{2}(x) T_{3}(x) (\pi^{2}-4 x^{2})}$, where $H_{7}(x)=378\mbox{,}675 (\pi^{4}-112 \pi^{2}+1008) \pi^{2} + (-64\mbox{,}350 \pi^{6}+5\mbox{,}536\mbox{,}440 \pi^{4}+106\mbox{,}323\mbox{,}840 \pi^{2}-1\mbox{,}526\mbox{,}817\mbox{,}600)x^{2} + (1968 \pi^{6}+50\mbox{,}400 \pi^{4}-25\mbox{,}764\mbox{,}480 \pi^{2}+ 247\mbox{,}484\mbox{,}160) x^{4} + (-8008 \pi^{4}+820\mbox{,}512 \pi^{2}-7\mbox{,}318\mbox{,}080)x^{6}$. By using the Maple software, $H_{7}(x)$ has six real roots $\approx -9.16,-4.97 ,-2.76, 2.76, 4.97, 9.16$, and $H_{7}(x), T_{2}(x), T_{3}(x)> 0, \forall x \in (0,1.5701)$, we have that

$$c_{6}(x) - b_{6}(x) \leq 0,\quad \forall x \in [0,1.5701]. $$
